# Schwannoma of the membranous nasal septum

**DOI:** 10.5935/1808-8694.20130140

**Published:** 2015-10-08

**Authors:** Joginder Singh Gulia, Samarpal Singh Yadav, Sukhdeep Kaur Basur, Anita Hooda

**Affiliations:** a Professor (Department of Otorhinolaryngology, Pt. B.D Sharma University of Health Sciences Rohtak, Haryana, India); b Senior Professor (Department of Otorhinolaryngology, Pt. B.D Sharma University of Health Sciences Rohtak, Haryana, India); c Postgraduate student (Department of Otorhinolaryngology, Pt. B.D Sharma University of Health Sciences Rohtak, Haryana, India); d Associate Professor (Department of Oral Anatomy, Pt B.D Sharma University of Health Sciences Rohtak, Haryana, India); Department of Oral Anatomy, Pt B.D Sharma University of Health Sciences Rohtak, Haryana, India

**Keywords:** nasal septum, nerve sheath neoplasms, neurilemmoma

## INTRODUCTION

Schwannoma is a benign tumor of nerve sheath of any myelinated nerve. The most frequent site affected in the head and neck is the eighth cranial nerve, other sites include: scalp, face, oral cavity, pharynx, larynx, trachea, parotid gland, middle ear and external auditory meatus. Schwannomas almost always occur as solitary lesions^1^. Tumors arising from the membranous part of the septum are very rare. A PubMed search (till June 2013) including the keywords schwannomas, neurilemmoma, and membranous septum retrieve only one report^2^. A case of schwannomas arising from the membranous part of the nasal septum presenting as a cystic swelling is reported.

## CASE PRESENTATION

A 35 year old female patient presented with 10 month history of swelling in the left nasal cavity, which slowly increased in size. She complained of pain and unilateral (left sided) nasal obstruction. There was no history of epistaxis, facial numbness, anosmia or facial swelling. Past history was insignificant.

**Figure 1:**
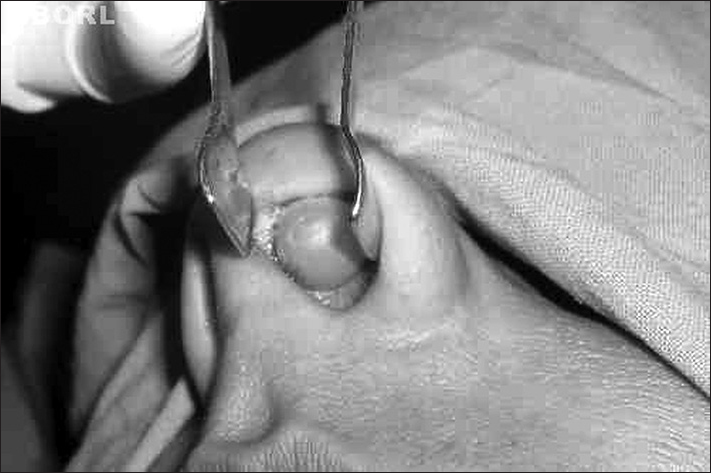
Showing a smooth cystic swelling of approximate size 1.5 × 1.0 cm on the left side of the membranous nasal septum.

General physical examination was normal. Otorhinolaryngological examination of the nasal cavity revealed a smooth, cystic swelling of approximate size 1.5 × 1.0 cm on the left side of the membranous nasal septum. On palpation it was soft, painless, and slightly mobile (Figure 1).

The routine radiological and blood investigations were normal. After anesthetic fitness the patient was taken up for surgery under local anesthesia. The swelling was excised. The excised specimen was yellow colored tumor with a soft consistency measuring 1.5 × 1.0 × 1.0 cm. Histopathological examination showed Antoni A pattern with nuclear pallisading, confirming the diagnosis of schwannoma. Postoperative period was uneventful and after a follow up of 6 months, patient has no recurrence.

## DISCUSSION

Schwannomas are lesions that arise from the neural sheath of peripheral, autonomic, or cranial nerves. Nerve sheath tumors of the head and neck region mainly involve the eighth cranial nerve with only 4% occurring in the paranasal sinuses. Preferential locations have been reported as being the ethmoid sinuses, followed by the maxillary sinus, the nasal pits, and the sphenoid sinus3., 4.. Localization to the membranous part of the nasal septum is rare^2^.

Nasal schwannomas mostly occur between the ages of 20 to 60 years with no sex or racial predilection. In nasal vestibule they usually present with unilateral nasal obstruction, epistaxis, pain, and occasionally localized facial numbness. The tumor is slow growing, and reaches a considerable size; however nasal vestibular schwannomas tend to become symptomatic earlier and are comparatively smaller in size at presentation. As a result, they are usually excised without the need for radiologic imaging4., 5..

Macroscopically, schwannomas appear as gelatinous or cystic, well encapsulated masses. Microscopically, schwannomas are classified into two types. Antoni A is characterized by areas of high cellularity with spindle shaped cells, often arranged in bundles, palisades, or whirls. Groups of compact parallel nuclei are also seen and are known as “verocay bodies”. Antoni B is typified by loose myxoid stroma with spindle cells running in a haphazard manner^4^.

The differential diagnosis of a nasal tumour includes a wide variety of pathology including inflammatory polyps, juvenile angiofibroma, inverted papilloma, meningioma, neurofibroma, melanoma and olfactory neuroblastoma^6^.

Treatment is surgical excision, the surgical approach is determined according to the location and extent of the lesion. Recurrences are rare.

## CLOSING REMARKS

Schwannoma arising as a cystic mass from the membranous nasal septum is very rare. This case adds nasal Schwannoma to the differential diagnosis of cystic lesions of this area.

## References

[1] Butugan O, Grasel SS, de Almeida ER, Miniti A. (1993). Schwannoma of the nasal septum. Apropos of 2 cases. Rev Laryngol Otol Rhinol (Bord).

[2] Gul E, Piechnik-Resler D, Gul A. (2005). A case of schwannoma of the membranic part of nasal septum. Otolaryngol Pol.

[3] Sharma R, Tyagi I, Banerjee D, Pandey R. (1998). Nasoethmoid schwannoma with intracranial extension. Case report and review of literature. Neurosurg Rev.

[4] Donnelly MJ, al-Sader MH, Blayney AW. (1992). Benign nasal schwannoma. J Laryngol Otol.

[5] Ling L, Chen HH, Zhou SH, Teng XD, Lu YY. (2006). Neurilemmomas of the nasal vestibule: report of two cases. Chin Med J (Eng).

[6] Kaufman SM, Conrad LP. (1976). Schwannoma presenting as nasal polyp. Laryngoscope.

